# Pre-Operative Risk Factors Predict Post-Operative Respiratory Failure after Liver Transplantation

**DOI:** 10.1371/journal.pone.0022689

**Published:** 2011-08-01

**Authors:** Ching-Tzu Huang, Horng-Chyuan Lin, Shi-Chuan Chang, Wei-Chen Lee

**Affiliations:** 1 Department of Respiratory Therapy, Chang Gung Memorial Hospital, Taoyuan, Taiwan; 2 Institute of Emergency and Critical Care Medicine, National Yang-Ming University, Taipei, Taiwan; 3 Department of Thoracic Medicine, Chang Gung Memorial Hospital, and Department of Chinese Medicine, Chang Gung University College of Medicine, Taoyuan, Taiwan; 4 Chest Department, Taipei Veterans General Hospital, Taipei, Taiwan; 5 Department of General Surgery, Chang Gung Memorial Hospital, Chang Gung University College of Medicine, Taoyuan, Taiwan; University of Colorado Denver, United States of America

## Abstract

**Objective:**

Post-operative pulmonary complications significantly affect patient survival rates, but there is still no conclusive evidence regarding the effect of post-operative respiratory failure after liver transplantation on patient prognosis. This study aimed to predict the risk factors for post-operative respiratory failure (PRF) after liver transplantation and the impact on short-term survival rates.

**Design:**

The retrospective observational cohort study was conducted in a twelve-bed adult surgical intensive care unit in northern Taiwan. The medical records of 147 liver transplant patients were reviewed from September 2002 to July 2007. Sixty-two experienced post-operative respiratory failure while the remaining 85 patients did not.

**Measurements and Main Results:**

Gender, age, etiology, disease history, pre-operative ventilator use, molecular adsorbent re-circulating system (MARS) use, source of organ transplantation, model for end-stage liver disease score (MELD) and Child-Turcotte-Pugh score calculated immediately before surgery were assessed for the two groups. The length of the intensive care unit stay, admission duration, and mortality within 30 days, 3 months, and 1 year were also evaluated. Using a logistic regression model, post-operative respiratory failure correlated with diabetes mellitus prior to liver transplantation, pre-operative impaired renal function, pre-operative ventilator use, pre-operative MARS use and deceased donor source of organ transplantation (*p*<0.05). Once liver transplant patients developed PRF, their length of ICU stay and admission duration were prolonged, significantly increasing their mortality and morbidity (*p*<0.001).

**Conclusions:**

The predictive pre-operative risk factors significantly influenced the occurrence of post-operative respiratory failure after liver transplantation.

## Introduction

Liver transplantation (LT) is currently the only definite treatment for acute liver failure and chronic end-stage liver diseases. Because of a shortage of liver donations, patients may have to wait for a long time for a liver transplantation. When liver transplantations are performed, the patients are already very sick. These patients may also have a high incidence of common respiratory disorders including atelectasis, pleural effusion and poor compliance of the respiratory system due to edema of the chest wall or high intra-abdominal pressure. All of these respiratory disorders can affect the function of alveolar gas exchange. Some patients may even need intubation and ventilation.

Liver transplantation is an upper abdominal surgery which involves an extensive operation field and a long operation time. The surgical wound transects the abdominal oblique muscles and rectus muscles which are usually associated with respiratory movements [1]. Patients undergoing upper abdominal surgery are prone to diaphragmatic dysfunction which results in a 50–60% reduction in vital capacity and 30% reduction in functional residual capacity [2], [3]. In addition, the usage of anesthetics and the inhibitory effect of wound pain on coughing and mucous removal usually contribute to the development of post-operative pulmonary complications. In the literature, 5–10% of patients with general surgery develop post-operative pulmonary complications, especially in the patients with abdominal surgery [4]. Glanemann *et al.* [1], [5] observed that 11% of liver transplantation patients required ventilator assistance after transplantation and 36.1% required re-intubation. Among the patients who developed pulmonary complications and needed re-intubation, 44.6% of the patients were intubated within 24 hours after liver transplantation. All of these pulmonary complications contribute to a significant reduction in short-term survival.

Post-operative respiratory failure (PRF) [6], [7] is one of the most common post-operative pulmonary complications and may result in mortality. Pre-transplant risk factors that affect mortality and morbidity after liver transplantation have been investigated. However, the impact of PRF on LT patients' prognosis is still unclear.

The objective of this study was to identify which pre-transplant risk factors are likely to cause PRF.

## Results

### Patients

A total of 147 liver transplant patients, 113 males and 34 females, were included in this study. The average age of these patients was 50.2±8.7 years. The most common indication for liver transplantation was liver cirrhosis (76.2%), followed by fulminate hepatic failure (14.3%) and hepatocellular carcinoma (8.8%). There was no significant difference regarding total ischemic time (41.6±11.4 minutes vs. 39.0±10.4 minutes, p = 0.40, including cold and warm ischemic time) and duration of surgery (12.9±2.1 hours vs. 14.2±2.1 hours, p = 0.97). The demographic characteristics of the patients are shown in Table 1. Pre-operative co-morbidities included diabetes mellitus in 15% of patients, impaired renal function in 17.7%, and ventilator usage in 10.2%. Pre-operative pulmonary function tests showed restrictive defects in 17.7% of the patients. According to the Taiwan Organ Registry and Sharing Center, the model for end-stage liver disease (MELD) score is divided into three categories, 10–18, 19–24, and ≥ 25, which accounted for 35.4%, 28.6%, and 36.1% of the patients, respectively.

**Table 1:** Demographic data of the study subjects.

Demographic (n = 147)	Mean± SD (range)/number(%)
Gender, Male/Female	113(76.9)/34(23.1)
Age	50.2±8.7(45)
Comorbidities	
Diabetes Mellitus	22(15.0)
Heart disease	4(2.7)
Hypertension	7(4.8)
Renal insufficiency	26(17.7)
Ventilation required pre-transplantation	15(10.2)
Pulmonary function test, restrictive defects	26(17.7)
MARS	11(7.5)
Child-Turcotte-Pugh score	
B	30(20.4)
C	117(79.6)
MELD	
10–18	52(35.4)
19–24	42(28.6)
≧25	53(36.1)
Donor Group	
Living donor liver transplantation	93(63.3)
Deceased donor liver transplantation	54(36.7)

Abbreviations: MARS, Molecular adsorbent recycling system; MELD, Model for end-stage liver disease score.

### Univariate analysis

Among the 147 patients, 62 (42.2%) patients developed PRF and 85 (57.8%) did not (Table 2). Among the 62 patients with PRF, 14 (22.6%) required ventilation to support gas exchange pre-operatively, and 32 (51.6%) required re-intubation after operation. Among the 85 patients without PRF, only 1 (1.2%) required pre-operative ventilation to support gas exchange (*p*<0.001). There was no difference in age or sex between the two groups. The etiology of liver disease in both groups was different (*p* = 0.020). The PRF group had more patients with fulminant liver failure than the non-PRF group. There was a significant difference in MELD categories between these two groups (*p* = 0.004). Thirty (48.4%) PRF group patients had a MELD score ≥ 25 while only 23 (27.1%) of the non-PRF group patients had a MELD score ≥ 25. The severity of diseases was higher in the PRF group than in the non-PRF group.

**Table 2:** Pre-operative clinical parameters of the patients who underwent liver transplantation (n = 147), by univariate analysis.

Parameter	Postoperative respiratory failure	No postoperative respiratory failure	*p* value
	(n = 62)	(n = 85)	
	Mean ± SD/number(%)	Mean± SD/number(%)	
Age, years	50.2±8.5	50.2±8.9	.995
Gender, Male	47(75.8%)	66(77.6%)	.794
Etiology			.020
Liver cirrhosis	47(75.8%)	65(76.5%)	
Hepatocellular carcinoma	2(3.2%)	12(14.1%)	
Fulminate hepatic failure	13(21.0%)	8(9.4%)	
Comorbidities			
Diabetes Mellitus	14(22.6%)	8(9.4%)	.027
Heart disease	2(3.2%)	2(2.4%)	1.000
Hypertension	5(8.1%)	2(2.4%)	.133
Renal insufficiency	19(30.6%)	7(8.2%)	<.001
Ventilator required pre-transplantation	14(22.6%)	1(1.2%)	<.001
MARS	9(14.5%)	2(2.4%)	.009
Pulmonary function test			.008
Restrictive defects	17(27.4%)	9(10.6%)	
Donor Group			.004
Living donor liver transplantation	31(50.0%)	62(72.9%)	
Deceased donor liver transplantation	31(50.0%)	23(27.1%)	
MELD			.004
10–18	13(21.0%)	39(45.9%)	
19–24	19(30.6%)	23(27.1%)	
≧25	30(48.4%)	23(27.1%)	
Child-Turcotte-Pugh score			.054
Class B	8(12.9%)	22(25.9%)	
Class C	54(87.1%)	63(74.1%)	

Abbreviations: MARS, Molecular adsorbent recycling system; MELD, Model for end-stage liver disease score.

Pre-operative pulmonary function tests showed 27.4% restrictive defects in the PRF group, which was higher than in the non-PRF group (*p* = 0.008). Pre-operative co-morbidities including diabetes mellitus and renal function insufficiency were also higher in the PRF group than in the non-PRF group. Moreover, more patients in the PRF group than in the non-PRF group required MARS while waiting for liver transplantation (p = 0.009). For operation type, patients in the PRF group had a higher rate of deceased donor liver transplantation than patients in the non-PRF group (p = 0.004). All of the deceased donors were brain-dead donors.

### Multivariate analysis

To determine the independent factors between these two groups, all significant factors in univariate analysis were further analyzed by logistic regression. The results showed that the risk factors for PRF were diabetes mellitus, impaired renal function, pre-operative ventilator support, usage of MARS, and deceased donor liver transplantation (Table 3).

**Table 3:** Pre-operative predictors of post-operative respiratory failure by multivariate analysis.

Parameter	*p* value	Adjusted odds ratio (95% CI)
Diabetes mellitus	.001	7.55(2.28, 25.02)
Mechanical ventilation pre-transplantation	.002	38.85 (3.78, 398.96)
Renal insufficiency	.003	5.93(1.82, 19.35)
Deceased donor	.006	3.44(1.42, 8.38)
MARS	.024	14.09(1.42, 139.69)
MELD	.152	2.21(0.75, 6.50)
Restrictive defects	.728	0.78(0.19, 3.11)
Etiology	.081	0.51 (0.24, 1.09)

Abbreviations: MARS, Molecular adsorbent recycling system; MELD, Model for end-stage liver disease score.

**Figure 1:**
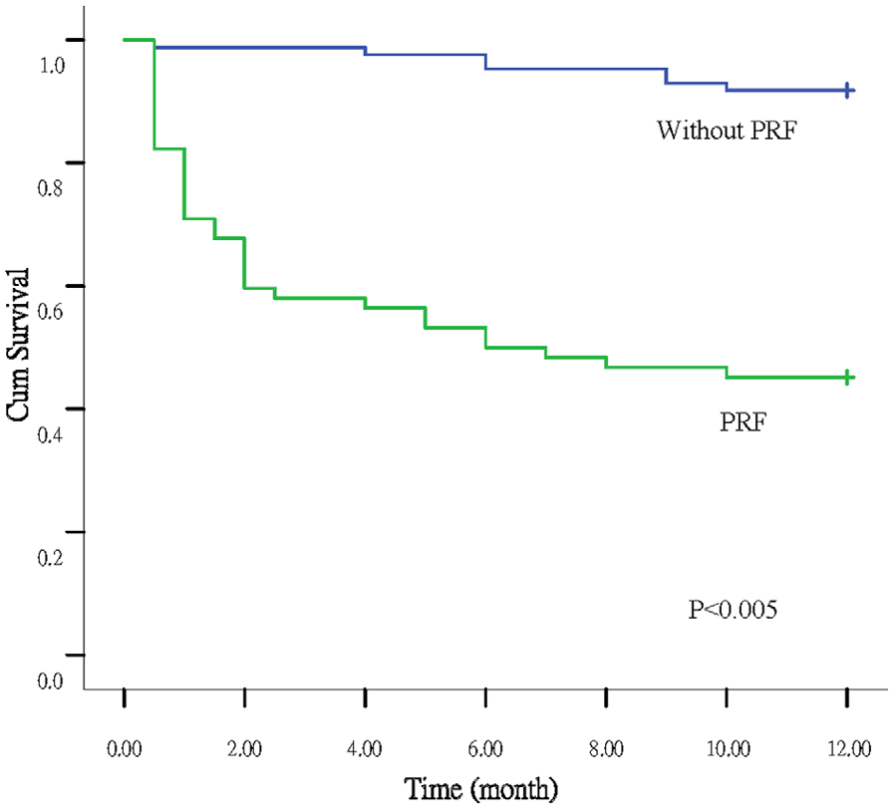
The survival rate for patients with or without PRF by Kaplan Meier analysis.

Once PRF developed, significant differences in post-operative prognosis were observed in both groups (Table 4). The length of ICU stay and duration of hospitalization were both longer in the PRF group than in the non-PRF group. Thirty-day, three-month, and one-year mortality rates were higher in the PRF group than in the non-PRF group. Kaplan-Meier survival curves showed that the survival rate at one year was 43.5% for the patients with PRF and 90.6% for the patients without PRF (*p*<0.001) (Fig. 1). A total of 43 patients died during the one-year study period (Table 4). The causes of death of 35 PRF patients included sepsis with multiorgan failure (29 patients), rejection (2), gastrointestinal hemorrhage (2), cardiac dysfunction (arrhythmia, 1), and pulmonary embolism (1). All the deaths in non-PRF group were due to sepsis with multiorgan failure (8 patients).

**Table 4:** Pre-operative predictors of short-term morbidity and mortality rate.

Parameter	Postoperative respiratory failure	No postoperative respiratory failure	*p* value
	(n = 62)	(n = 85)	
	Median (IQR)/number (%)	Median (IQR)/number (%)	
ICU stay, d	27 (6, 152)	9 (1, 65)	<.001
Hospital stay, d	51 (6, 231)	32 (5, 156)	<.001
Mortality (30-days)	23 (37.1)	1 (1.2)	<.001
Mortality (three-months)	28 (45.2)	1 (1.2)	<.001
Mortality (one-year)	35 (56.5)	8 (9.4)	<.001

Abbreviations: ICU stay, intensive care unit stay.

There were 15 (10.2%) patients who required ventilator support before transplantation. According to the medical records, there were no preoperative ventilator associated pneumonias in our patient population.

## Discussion

This retrospective study showed that pre-operative ventilator support, diabetes mellitus, impaired renal function, and deceased transplant recipients were all pre-operative risk factors for PRF. Once PRF developed, the length of stay at the intensive care unit and total duration of hospitalization both increased and caused a significant impact on short-term mortality after liver transplantation. Liver transplantation, compared with heart and kidney transplantations, are particularly prone to PRF and acute pulmonary damage. Previous study [9] had mentioned about “the risk of respiratory failure and acute lung injury is considerably lower after heart and kidney transplantation than liver transplantation”. Only 4.4% heart transplantation patients require tracheostomy for the development of prolong respiratory failure. Similarly, perioperative respiratory failure was documented in 4% recipients of kidney transplantation. Once PRF develops, both patient prognosis and survival rate are affected [9]. Glanemann *et al*. [5] reported that of 546 liver transplantation patients, 11% needed ventilator support for more than 24 hours, and 14.8% underwent extubation within 24 hours but required re-intubation later on. The patients in need of re-intubation have significantly reduced survival rates [1]. Arozullah *et al*. [7] adopted a prospective cohort model to predict the multi-factorial risk index for PRF after major noncardiac surgery. They discovered that 37% of liver transplant patients developed PRF and were unable to undergo extubation, while 29% of the patients who developed PRF required re-intubation. For those patients who were unable to undergo extubation, the mortality rate increased to 23% within 30 days. For those patients who were re-intubated, the mortality rate increased to as high as 31% within 30 days. Golfieri *et al*. [10] also described that 4–16% of patients who developed pulmonary complications after liver transplantation deteriorated into acute respiratory distress syndrome with a mortality rate as high as 80–100%. Clearly therefore, patients suffering from PRF after transplantation have a higher incidence of short-term mortality.

Glanemann *et al*. [11] special attention should be focused on liver transplant recipients in poor clinical condition at the time of orthotopic liver transplantation, undergoing complicated surgery, or receiving liver grafts with severe preservation injury. Our work provides a worthwhile scientific study on the field of risk factors for post-operative respiratory failure after liver transplantation and the impact on short-term survival rates. Postoperative respiratory failure is one of the most common post-operative pulmonary complications and may result in mortality [12]. González *et al.* [12] suggested that the development of acute respiratory failure after liver transplantation is affected by the following factors: female sex, Child-Pugh class, pulmonary edema, postoperative acute renal failure, cerebral dysfunction, and respiratory infection. However, only few studies have addressed the impact of pre-transplantation risk factors on the post-operative respiratory failure after liver transplantation. Besides, pre-transplantation risk factors that affect mortality and morbidity after liver transplantation have been investigated. For example, Preeti JR *et al.* [13] reported that preexisting diabetes is associated with a significant post-orthotopic liver transplantation morbidity and mortality. However, the impact of post-operative respiratory failure on liver transplantation patients' prognosis is still unclear. Therefore, our paper will provide comprehensive and potential information for clinical physician to improve the critical care for these patients.

Multisystem organ failure (MSOF) is important for liver transplantation patients. In our study, liver transplantation patients were tightly monitored once they are on the waiting list. Multisystem organ failure occurred before surgery is not suitable for liver transplantation. Actually, in our previous study [14] entitled “Scoring Short-Term Mortality After Liver Transplantation”, we used Sequential Organ Failure Assessment (SOFA) score for measuring multiple organ failure for patients and found that SOFA scores calculated before liver transplantation were statistically significant predictors of 3-month and 1-year posttransplant mortality. However, only SOFA on post–liver transplant day 7 had the best discriminative power for predicting 3-month and 1-year mortality after liver transplantation. Interestingly, in our study, we found that the PRF on post-liver transplant day 2 was associated with a higher SOFA score on post-liver transplant day 7, compared with those in patients without PRF (8.1±3.4 vs. 4.9±1.8, p<0.001). It suggested that PRF on post–liver transplant day 2 is an earlier predictor for the outcome than the SOFA score as described in the previous study.

There was a higher rate of post-operative respiratory failure in our population. It might be a matter of local differences in anesthetic and ICU management, or differences in patient or donor graft characteristics compared to other reports. We had reviewed the previous studies on early extubation in liver transplant recipients and found that there are some discrepancies in the patient's enrollment. The exclusion criteria in the literature on early extubation in transplant recipients included acute hepatic failure, ventilator required pretransplantion [15] and living donors [16]. However, in our study, we did not exclude the patients mentioned above. In addition, given the limited source of organ donors, the waiting time for prospective liver transplantation is long, making it difficult to control the disease severity. Concerning the donor group in our data, there is 36.7% patients received deceased donors. Obviously, deceased donors group had higher MELD score (25.6±8.4) compared with living donors and deceased donor liver transplantation belongs to the urgent surgery. While comparing the severity of liver disease before surgery, patients with Child's class C occupied 79.6%, which was higher than that in previous study [17], [18]. Those are the reasons why there is a higher prevalence of respiratory failure in our population. The outcomes differ among institutions and dependent upon experience, resources and the patient population. While the description of respiratory failure in their patient population is of some interest, most readers will reject the data if it is not similar to their own experience. It is very apparent that there are a number of peer reviewed publications with different outcomes. However, the one thing that is applicable to all centers is the systematic study of risk factors for each institution.

Impairment of renal function is an independent risk factor for the development of post-operative pulmonary complications. In this study, pre-operative impairment of renal function between the PRF and non-PRF patients was significantly different. Multivariate analysis also showed that impaired renal function was an independent factor with an adjusted odds ratio of 5.93 (95% CI, 1.82–19.35; *p* = 0.003). All the patients in our study with preoperative renal insufficiency did not require preoperative dialysis, as well as intraoperative dialysis or ultrafiltration. Impaired renal function with an imbalanced pH value increases the work of breathing and reduces pulmonary compliance. Once respiratory failure occurs and mechanical ventilation is adopted, the high intra-thoracic pressure will affect the systemic and renal hemodynamics leading to a drop in cardiac output that will in turn affect renal blood flow [19]. Nair *et al*. [8] showed that pre-operative serum level of creatinine (>1.5 mg/dl) was an important indicator for assessing post-operative ICU stay as well as short-term survival rate [20], [21], [22]. These results imply that it is better to perform liver transplantation before renal function becomes impaired. In our study, we did not perform combined liver kidney transplantation in our renal failure patients.

Diabetes mellitus patients are prone to have delayed wound healing after major surgery and an increased risk of infection and morbidity. The results in this study showed that patients suffering from diabetes mellitus prior to surgery had a higher chance of developing PRF after surgery. The hazard ratio for diabetes mellitus was 7.55 (95% CI, 2.28–25.02; *p* = 0.001). Immunosuppressive agents such as tacrolimus and steroids may influence the metabolism of glucose. Impaired renal function and gastric emptying may both interfere with blood levels of immunosuppressants and ultimately lead to poor blood glucose control and infections. Preeti *et al*. [13] discovered that compared to non-diabetic patients, diabetic patients had a significantly higher serum creatinine level prior to liver transplantation and a higher incidence of pulmonary complications after transplantation (*p* = 0.001).

The usage of MARS was a risk factor to develop post-operative PRF. Eleven patients in the current study received MARS combined with dialysis treatment. Of these 11 patients, 9 (81.8%) developed PRF, which was significantly higher than in the patients who did not receive MARS (*p* = 0.009). Using an artificial liver/biological artificial liver as a support system to extend the waiting time increases the opportunity of liver transplantation for acute liver failure patients. Most toxins produced by liver failure bind to albumin, and traditional hemodialysis cannot effectively remove the toxicity for acute liver failure patients. Non-biologic artificial liver support therapies, MARS, combine a molecular adsorbent re-circulating system and a dialysis system to remove water-soluble and protein-bound toxins. The mortality rate within one week has been shown to be 100% and 63% for the control group and the MARS-treatment group, respectively [23]. Although MARS treatment extends the waiting time for liver transplantation and possibly improves the survival rate for the patients with hepato-renal syndrome, the usage of MARS is still a risk factor to develop PRF.

According to previous report, the criteria of MARS including acute decompensation on chronic liver disease, acute liver failure, primary graft dysfunction, liver failure post-liver surgery and intractable pruritus in chronic cholestatic syndromes [24]. The waiting time for prospective liver transplantation is long, making it difficult to control the disease severity. In aid of extending the waiting time, 11 patients (7%) in our study received MARS to increase the opportunities of liver transplantation for acute decompensation on chronic liver disease and acute liver failure patients requiring intubation.

The patients were divided into three groups according to a MELD score of 10–18, 19–24, and ≥ 25 in this study. Thirty among 62 patients (48.4%) with PRF had a MELD score ≥ 25, compared to 23/85 (27.1%) patients in the non-PRF group. Among all 53 patients with a MELD score ≥ 25, the incidence of PRF was 56.6% (30/53), compared to 45.2% (19/42) for the patients with a MELD score between 19 to 24 and 25% (13/52) for the patients with a MELD score between 10 to 18. These results implied that the patients with a high MELD score had a higher incidence of PRF. Saab *et al*. [25] reported that the one-year survival rate was significantly different when the patients were divided into MELD scores < 24 and > 24. Previous studies have noted that MELD score could more accurately predict ventilator usage for gas exchange support in liver pre-transplantation than the CTP score, however, it could not be used to predict short-term survival rate [26], [27], [28], [29]. In this study, MELD score was not an independent factor to predict PRF, however, the patients with high MELD scores may have had a higher rate of comorbidities.

In previous study [30], patients were initially stratified into 7 groups based on the MELD score of <10, 11–15, 16–20, 21–25, 26–30, 31–35, and >36. Graft and patient survival were compared among the groups. Groups with similar results were merged to develop 3 larger categories as defined by pretransplantation MELD of <15 (low risk), 16–25 (medium risk), and >25 (high risk). It is consistent with our stratification for the pretransplant MELD scores. Based on this MELD scoring system, patients are equally distributed in the three categories and are suitable for analysis. In our study, it did show that a MELD score of 25–40 is significantly associated with a higher rate of PRF, compared with those in other groups.

Feng *et al.* [31] suggested that the donor risk index (DRI), calculated by eight-parameter formula, was an important predictor of graft failure. In another cohort study, Maluf *et al*. [32] found that a DRI of more than or equal to 1.7 is a cutoff value in defining an extended criteria for donor graft. In our study, the DRI between non-PRF and PRF patients was not significantly different (1.423±0.210 and 1.499±0.342; p = 0. 578), both were less than 1.7, suggesting that DRI may not a predictor of post-operative respiratory failure in our patient population.

Intraoperative care is also an important issue. Actually, we found that there was significant difference in the perioperative blood loss between non-PRF and PRF patients (3954±3921 ml and 6657±6566 ml; p = 0.013). More patients in non-PRF group completed the surgical procedure without the need for blood transfusion compared with the PRF group. We believe that perioperative care of transplant recipients should be an important predictor of outcome. However, our present study highlights the role of pre-operative risk factors. It needs further study to investigate the perioperative risk factors associated with PRF.

Previous papers have found encephalopathy, massive transfusion requirements, primary graft failure, cardiac failure, multiorgan transplant, and retransplantation were all contraindications to early (<24 h) extubation after liver transplantation. However, in our study population, there were no patients who were primary graft failure, cardiac failure, multiorgan transplant, and retransplantation. Similarly, no patients had required massive transfusion before transplantation. In our study, encephalopathy was a fluctuated factor that is difficult to evaluate from the medical record. It needs further study to investigate the indicator.

In our study, 93 (63.3%) patients received living donor liver transplantation (Table 1). Previous studies [16] have suggested living donation is a contraindication to early extubation. However, in our data, we found that living donor liver transplantation had a lower rate (31/93, 33.3%) of PRF, in contrast with deceased donor liver transplantation (31/54, 57.4%)(Table 2). Our finding is interesting and provides a potential therapeutic direction for clinical practice.

There were significant differences in ICU stay after surgery, hospital stay, 30-day mortality, three-month mortality, and one-year mortality between patients with or without PRF. Kaplan-Meier survival analysis showed that the prognosis for the patients with or without PRF was also significantly different.

Although transplantation is effective, the possibility of transplantation depends on the availability of a liver donor. Therefore, predictors of mortality risk and models for the short term prognosis of end-stage liver disease are needed to help clinicians and policymakers predict the outcomes of liver transplantation. In our previous study [14], we found that among 4 evaluated scoring systems: (1) The Sequential Organ Failure Assessment (SOFA) score, (2) Child-Pugh points, (3) Model for End-Stage Liver Disease score, and RIFLE (risk of renal dysfunction, injury to the kidney, failure of the kidney, loss of kidney function, and end-stage kidney disease) criteria, only the SOFA scores calculated before liver transplantation were statistically significant predictors of 3-month and 1-year posttransplant mortality. SOFA on post–liver transplant day 7 had the best discriminative power for predicting 3-month and 1-year mortality after liver transplantation. Moreover, Preoperative hyponatremia was also a significant risk for postoperative complications and short-term graft loss [33]. The addition of sodium concentration to MELD score might therefore be an effective predictor for post-transplant short-term mortality in deceased donor liver transplantation. Older patient and donor age [34], male sex of recipient, retransplantation, and high pre-transplant MELD score are associated with poor post-transplant outcome [30]. Our study provided a new concept that post-operative respiratory failure is a key factor in liver transplant that carries prognostic impact in the recipients.

After liver transplantation, patients need to receive regular immunosuppressive treatment, compared with other surgical patients. It is supposed that patients would have a high infection opportunity. However, only few studies have addressed the impact of infection on the short-term mortality after liver transplantation. Better predictive models are needed to assess the infection associated short-term mortality.

### Conclusion

In conclusion, this study identified several pre-operative risk factors for PRF, which lead to a prolonged ICU and hospital stay and admission duration and affected morbidities and mortality. We recommend that ventilated patients should be weaned, and impaired renal function and coagulation function be well controlled prior to liver transplantation in order to reduce PRF and thereby improve outcomes.

## Materials and Methods

### Patients

The study design was a retrospective review of patient records with approval of Institutional Review Board (IRB), Chang Gung Medical Foundation (IRB no.: 97-0567B). Methodology and patient confidentiality were approved by our IRB. The IRB confirmed that this study constituted an audit, which did not require patient consents to this retrospective study. From September 2002 to July 2007, the medical records of 153 patients who had liver transplantations in Chang-Gung Memorial Hospital were reviewed. Six patients were excluded due to incomplete data collection. Therefore, 147 patients were included in this study.

### Definition of post-operative respiratory failure

In our ICU, a weaning protocol was followed to wean the ventilator after transplantation surgery. Briefly, weaning was started after reversal of neuromuscular function, under adequate of cardiovascular, respiratory and metabolic function, and the weaning criteria were fulfilled: rapid shallow breathing index or respiratory rate/tidal volume ratio ≤105 breaths/min/L (tidal volume>5 ml/Kg, frequency less than 30 breaths/min; maximum inspiratory pressure or negative inspiratory force less than - 30 cmH2O). The arterial blood gas analysis was within normal limit under FiO2≤0.4 and PaO2/FiO2 ratio>350. When the patient was stable and could maintain spontaneous breathing for 30–60 minutes, the surgeon and respiratory therapist determined whether an extubation should be performed in accordance with the above weaning criteria.

Post-operative respiratory failure (PRF) [6], [7] was defined as patients requiring ventilator support for more than 48 hours or patients having re-intubation. All 147 patients were divided into two groups: PRF patients, who developed post-operative respiratory failure, and non-PRF patients, who did not develop post-operative respiratory failure.

### Anesthetic regimen and early enteral feeding protocol

Short-acting anesthetic drugs were used as anesthetic regimen for our patients, including midazolam, fentanyl, and rocuronium that were administered on a dose per weight basis at induction. Anesthesia was maintained with an oxygen-air-isoflurane mixture and intermittent doses of cis-atracurium were given for continuing muscle relaxation. A standardized surgical technique performed by the same surgical team was used for all patients. The specific time for inferior vena cava clamping, portal venous reperfusion, and hepatic artery reperfusion was protocol-controlled to within 10 to 15 minutes. All patients were transferred to the ICU for post-transplantation care, including early enteral feeding protocol. Once patients exhausted, enteral feeding was started.

### Data collection

The data collected included patient profiles, etiology of diseases, history of systemic diseases (diabetes mellitus, hypertension, heart disease, or rental insufficiency), the definition of renal insufficiency as serum creatinine more than 1.5 mg/dL, or creatinine clearance (CCr) less than 70 mL/min following a previous report [8], pre-operative ventilator usage, model for end-stage liver disease score (MELD), Child-Turcotte-Push (CTP) Classification, pre-operative usage of molecular adsorbent re-circulating system (MARS), pre-operative pulmonary function tests (most recent pulmonary function (≤3 months) on file as relevant reference for liver transplantation), pre-operative laboratory data, length of intensive care unit (ICU) stay, and duration of hospitalization. The post-operative mortality within 30 days, three months, and one year were also collected.

### Statistical analysis

Data were analyzed using the statistical software package SPSS (Version 15 SPSS, Chicago, IL). Data were shown as mean ± SD, median with range, or percentages. The univariate relationship between each variable and PRF was tested using Pearson's chi-square or Fisher's exact tests. All significant variables in univariate analysis were analyzed by multiple regression logistic models. Overall patient survival was estimated using the Kaplan-Meier survival analysis. A *p* value < 0.05 was considered statistically significant.

## References

[1] Glanemann M, Kaisers U, Langrehr JM, Schenk R, Stange BJ (2001). Incidence and indications for re-intubation during post-operative care following orthotopic liver transplantation.. J Clin Anesth.

[2] Meyers J, Lembeck L, O'Kane H, Baue A (1975). Changes in functional residual capacity of the lung after operation.. Arch Surg.

[3] Smetana GW, Lawrence VA, Comell JE (2006). Preoperative pulmonary risk stratification for noncardiothoracic surgery: Systematic review for the American college of physicians.. Ann Intern Med.

[4] Wong DH, Weber EC, Schell MJ, Wong AB, Anderson CT (1995). Factors associated with postoperative pulmonary complications in patients with severe chronic obstructive pulmonary disease.. Anesthesia and Analgesia.

[5] Glanemann M, Langrehr J, Kaisers U, Schenk R, Müller AR (2001). Postoperative tracheal extubation after orthotopic liver transplantation.. Acta Anaesthesiol Scand.

[6] Svensson L, Hess K, Coselli J, Safi H, Crawford E (1991). A prospective study of respiratory failure after high-risk surgery on the thoracoabdominal aorta.. J Vasc Surg.

[7] Arozullah AM, Daley J, Henderson WG, Khuri SF (2000). Multi-factorial risk index for predicting post-operative respiratory failure in men after major non-cardiac surgery.. Ann Surg.

[8] Nair S, Verma S, Thuluvath PJ (2002). Pre-transplant renal function predicts survival in patients undergoing orthotopic liver transplantation.. Hepatology.

[9] Kotloff RM (2005). Non-infectious Pulmonary Complications of Liver, Heart, and Kidney Transplantation.. Clin Chest Med.

[10] Golfieri R, Giampalma E, Morselli Labate AM, d'Arienzo P, Jovine E (2000). Pulmonary complications of liver transplantation: radiological appearance and statistical evaluation of risk factors in 300 cases.. Eur Radiol.

[11] Glanemann M, Busch T, Neuhaus P, Kaisers U (2007). Fast tracking in liver transplantation. Immediate postoperative tracheal extubation: feasibility and clinical impact.. Swiss Med Wkly.

[12] González E, Galán J, Villalaín C, Valero JC, Silla I (2006). Risk factors for acute respiratory failure after liver transplantation.. Rev Esp Anestesiol Reanim.

[13] Preeti JR, Thuluvath PJ (2001). Outcome of liver transplantation in patients with diabetes mellitus: A case-control study.. Hepatology.

[14] Wong CS, Lee WC, Jenq CC, Tian YC, Chang MY (2010). Scoring short-term mortality after liver transplantation.. Liver Transpl.

[15] Findlay JY, Jankowski CJ, Vasdev GM, Chantigian RC, Gali B (2002). Fast track anesthesia for liver transplantation reduces postoperative ventilation time but not intensive care unit stay.. Liver Transpl.

[16] Mandell MS, Lezotte D, Kam I, Zamudio S (2002). Reduced Use of Intensive Care After Liver Transplantation: Influence of Early Extubation.. Liver Transpl.

[17] Mandell MS, Stoner TJ, Barnett R, Shaked A, Bellamy M (2007). A Multicenter Evaluation of Safety of Early Extubation in Liver Transplant Recipients.. Liver Transpl.

[18] Biancofiore G, Romanelli AM, Bindi ML, Consani G, Boldrini A (2001). Very early tracheal extubation without predetermined criteria in a liver transplant recipient population.. Liver Transpl.

[19] Kuiper JW, Johan Groeneveld AB, Slutsky AS, Plötz FB (2005). Mechanical ventilation and acute renal failure.. Crit Care Med.

[20] Baliga P, Merion RM, Turcotte JG, Ham JM, Henley KS (1992). Pre-operative risk factor assessment in liver transplantation.. Surgery.

[21] Ghobrial RM, Gornbein J, Steadman R, Danino N, Markmann JF (2002). Pre-transplant Model to Predict Post-transplant Survival in Liver Transplant Patients.. Ann Surg.

[22] Rodríguez-Ariza AM, Martínez-Galisteo E, Padilla CA, Bárcena JA, Fraga E (2008). Prognostic factors associated with postoperative complications in liver transplant.. Rev Esp Enferm Dig.

[23] Doria C, Mandala L, Scott V, Gruttadauria S, Marino IR (2006). Fulminant Hepatic Failure Bridged to Liver Transplantation with a Molecular Adsorbent Re-circulating System: A Single-Center Experience.. Digestive Dis Sci.

[24] Saliba F (2006). The Molecular Adsorbent Recirculating System (MARS®) in the intensive care unit: a rescue therapy for patients with hepatic failure.. Crit Care.

[25] Saab S, Ibrahim AB, Durazo F, Wang V, Han S (2003). MELD Score Predicts 1-Year Patient Survival Post- Orthotopic Liver Transplantation.. Liver Transpl.

[26] Brown RS, Shiva Kumar K, Russo MW, Kinkhabwala M, Rudow DL (2002). Model for End-Stage Liver Disease and Child-Turcotte- Pugh Score as Predictors of Pretransplantation Disease Severity, Post-transplantation Outcome, and Resource Utilization in United Network for Organ Sharing Status 2A Patients.. Liver Transpl.

[27] Narayanan Menona KV, Nyberga SL, Harmsenb WS, Rosena CB, Wiesnera RH (2004). MELD and Other Factors Associated with Survival after Liver Transplantation.. Am J Transpl.

[28] Farnsworth N, Fagan S, Berger D, Awad S (2004). Child-Turcotte-Pugh versus MELD score as a predictor of outcome after elective and emergency surgery in cirrhotic patients.. Am J Surg.

[29] Bilbao I, Armadans L, Lazaro JL, Hidalgo E, Castells L (2003). Predictive factors for early mortality following liver transplantation.. Clin Transpl.

[30] Habib S, Berk B, Chang CC, Demetris AJ, Fontes P (2006). MELD and prediction of post-liver transplantation survival.. Liver Transpl.

[31] Feng S, Goodrich NP, Bragg-Gresham JL, Dykstra DM, Punch JD (2006). Characteristics Associated with Liver Graft Failure: The Concept of a Donor Risk Index.. Am J Transplant.

[32] Maluf DG, Edwards EB, Kauffman HM (2006). Utilization of extended donor criteria liver allograft: Is the elevated risk of failure independent of the model for end-stage liver disease score of the recipient?. Transplantation.

[33] Fukuhara T, Ikegami T, Morita K, Umeda K, Ueda S (2010). Impact of preoperative serum sodium concentration in living donor liver transplantation.. J Gastroenterol Hepatol.

[34] Macedo FI, Miranda LE, Fernandes JL, Padua TC, Figueroa JN (2010). Donor age as a predictor of risk for short-term outcomes after liver transplant.. Experimental & Clinical Transplantation: Exp Clin Transplant.

